# *QuickStats:* Prevalence of High Total Cholesterol* Among Adults Aged ≥20 Years,^†^ by Age Group and Sex — National Health and Nutrition Examination Survey, 2015–2018

**DOI:** 10.15585/mmwr.mm6922a5

**Published:** 2020-06-05

**Figure:**
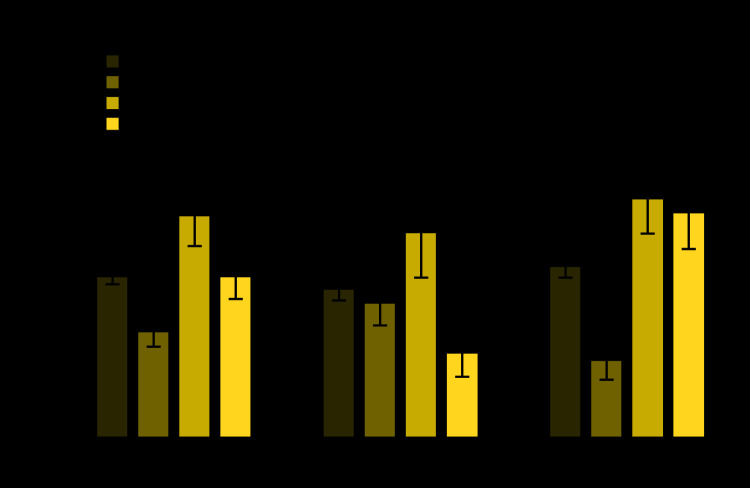
During 2015–2018, the prevalence of high total cholesterol among adults aged ≥20 years was 11.4%, with no significant difference between men (10.5%) and women (12.1%). Prevalence was highest among adults aged 40–59 years (15.7%), followed by those aged ≥60 years (11.4%), and lowest among those aged 20–39 years (7.5%). Among men, the prevalence was highest among those aged 40–59 years (14.5%), followed by those aged 20–39 years (9.5%), and lowest among those aged ≥60 years (6.0%). Among women, the pattern was different, with women aged 20–39 years (5.5%) having a lower prevalence than either women aged 40–59 years (16.9%) or women aged ≥60 years (15.9%). Prevalence among women aged 20–39 years was lower than that among men in this age group, but prevalence was higher among women aged ≥60 years than it was among men of that age group. There was no significant difference between men and women for adults aged 40–59 years.

**Sources**: Carroll MD, Fryar CD. Total and high-density lipoprotein cholesterol in adults: United States, 2015–2018. NCHS Data Brief, no 363. https://www.cdc.gov/nchs/products/databriefs/db363.htm. National Center for Health Statistics, National Health and Nutrition Examination Survey, 2015–2018. https://www.cdc.gov/nchs/nhanes.htm.

